# Ag85a-S2 Activates cGAS-STING Signaling Pathway in Intestinal Mucosal Cells

**DOI:** 10.3390/vaccines10122170

**Published:** 2022-12-16

**Authors:** Sheng Dang, Wanyang Li, Shubo Wen, Yang Song, Meirong Bai, Shuyan Li, Zeliang Chen, Jingbo Zhai

**Affiliations:** 1 Innovative Institute of Zoonoses, Inner Mongolia Minzu University, Tongliao 028000, China; 2 Keerqin District First People’s Hospital, Tongliao 028000, China; 3 Brucellosis Prevention and Treatment Engineering Research Center of Inner Mongolia Autonomous Region, Tongliao 028000, China; 4 Key Laboratory of Zoonose Prevention and Control at Universities of Inner Mongolia Autonomous Region, Tongliao 028000, China; 5 Key Laboratory of Mongolian Medicine Research and Development Engineering, Ministry of Education, Tongliao 028000, China

**Keywords:** *Brucella*, brucellosis, Ag85a

## Abstract

Brucellosis is a zoonotic disease caused by Gram-negative bacteria. Most of the brucellosis vaccines in the application are whole-bacteria vaccines. Live-attenuated vaccines are widely used for brucellosis prevention in sheep, goats, pigs, and cattle. Thus, there is also a need for an adjuvanted vaccine for human brucellosis, because the attenuated *Brucella* vaccines now utilized in animals cause human illness. Here, we developed a live-attenuated *Brucella suis* strain 2 vaccine (S2) adjuvanted with Ag85a (Ag85a-S2). We found that Ag85a-S2 activated cGAS-STING pathways both in intestinal mucosal cells in vivo and in the BMDM and U937 cell line in vitro. We demonstrated that the cGAS knockout significantly downregulated the abundance of interferon and other cytokines induced by Ag85a-S2. Moreover, Ag85a-S2 triggered a stronger cellular immune response compared to S2 alone. In sum, Ag85a-S2-mediated enhancement of immune responses was at least partially dependent on the cGAS-STING pathway. Our results provide a new candidate for preventing *Brucella* pathogens from livestock, which might reduce the dosage and potential toxicity compared to S2.

## 1. Introduction

Brucellosis, a zoonotic bacterial disease, is prevalent worldwide and in Inner Mongolia Autonomous Region (IMAR) in China [1,2]. The incidence of brucellosis and the number of patients infected by *Brucella* in IMAR rank first in China, and the Tongliao region is among the most-affected areas. Millions of sheep, cattle, pigs, and other livestock are infected annually in IMAR, causing a median loss of RMB 2 billion or USD 3.4 billion to the livestock sector in China or the USA [3,4]. *Brucella* typically infects people through the digestive tract, respiratory tract, and genitourinary mucosa through contact with infected dairy and meat products [1]. As one of the non-commensal pathogenic microorganisms, *Brucella* typically enters the organism through the intestinal mucosa and destabilizes the commensal biota. *Brucella* can cause various signs and symptoms, including fever, sweats, and malaise, some of which may present for prolonged periods. Upon *Brucella* infection, the host cells recognize pathogen-associated molecular patterns (PAMPs) of *Brucella* through pattern recognition receptors (PRRs) and activate innate/adaptive immune responses. On one hand, *Brucella* can initiate innate immune responses mediated by PRRs. Toll-like receptors (TLRs) are best characterized among the PRRs. According to previous literature, TLR2/4/9 were activated and engaged in bacterial signaling, host resistance, and dendritic cell activation [5,6]. On the other hand, according to immunological responses in human brucellosis, TH1 (interferon-producing T cells) are linked to dominant immune reactions, which is congruent with research in animals. Patients with persistent brucellosis have been reported as having abnormal THl response and anergy, which are linked to a poor prognosis [7].

Researchers have developed various vaccines to reduce the risk of *Brucella* infection. Most of the vaccines that produce significant protection effects are live-attenuated vaccines. *B. abortus* strains 19 and RB51, as well as *B. melitensis* strain Rev1, were widely used to prevent *Brucella* infection [8,9,10,11]. Although they provided a protective effect, they were not ideal. In animals, *Brucella* typically affects the reproductive organs of host animals, and abortion is often the only sign of the disorder [12]. The high virulence of the three vaccines mentioned above caused abortion in pregnant farm livestock and had potential pathogenicity in humans. In 1952, the Veterinary Drug Supervision Institute of the Ministry of Agriculture of China screened a naturally attenuated variant of *Brucella* from pig embryos [13]. Based on this finding, researchers made the *Brucella suis* S2 (S2) vaccine, officially used to immunize sheep and goats in the 1970s. Despite its use in sheep and goats, S2 is developed from a *Brucella suis* strain S2. Immunization can be administered orally or intramuscularly. The virulence of the S2 vaccine is low, and oral immunization outperforms conjunctival immunization [14]. Oral immunization of pregnant sheep, cattle, and pigs does not cause abortion [15]. However, the immunization dose of this vaccine per one sheep is recommended to be 20 billion units. Oral administration of such a large dose increases the potential risk of vaccine toxicity.

The Ag85a is a diacylglycerol acyltransferase involved in lipid body formation [16] and exists in the cell wall of Mycobacterium tuberculosis and the culture infiltrates of the *Bacille Calmette–Guérin* vaccine (BCG). Ag85a has a potent Th1-type cytokine-inducing potential [17]. Our previous studies found that Ag85a could effectively activate immune responses and improve patients’ immunogenicity [18,19,20,21,22]. Thus, it is quite possible that Ag85a could be used as an immune modulator to boost immunity in response to pathogen infection.

When certain pathogens infect host cells, DNA released from pathogens is sensed by the host cells, thereby activating the innate immune responses. One of these sensors was the cyclic GMP-AMP synthase in the cytoplasm (cGAS) [23]. After cGAS was bound to dsDNA from microbes, it dimerized and became an activated state, which then catalyzed the cyclization of ATP and GTP to form the second messenger, 2′3′-cyclic-GMP-AMP (cGAMP). The cGAMP, in turn, can be recognized by the cyclic dinucleotide sensor stimulator of interferon genes (STING) [24,25]. STING is an ER-associated membrane protein that is in an autoinhibitory status under the resting state [25]. The binding of cGAMP changed STING’s conformational and transported STING from the ER to the Golgi apparatus, activating TANK-binding kinase 1 (TBK1). Next, TBK1 phosphorylated the transcription factor interferon regulatory factor 3 (IRF3) [26]. Phosphorylated IRF3 dimerized, entered into the nucleus, and bound to the promoters of type I interferon, inflammatory factors, and chemokines, which then triggered the expression of antiviral/bacterial factors [27]. The downstream cytokines regulated by cGAS-STING, especially type I IFNs, typically lead to induction of an antimicrobial state, activation of adaptive immunity, and eventual clearance of the infection [28]. Intriguingly, the activation of STING has proven to engage in the protection against *Brucella* infection [29]. On the other hand, *Brucella* also promotes proteasome-mediated cGAS degradation to evade immune monitor [30]. Thus, the activation of cGAS-STING signaling may be linked to better protection against *Brucella*.

To address the large dosage concern of the S2 vaccine, we introduced an immunologic adjuvant of Ag85a into the wild-type *B. suis* S2 (named Ag85a-S2 hereafter). Ag85a-S2 significantly upregulates several components of the cGAS-STING signaling pathway in vivo and in vitro. We further generated a cGAS-KO cell line and demonstrated that the upregulation of several immune cytokines induced by Ag85a-S2 depended on cGAS-STING. Moreover, Ag85a-S2 treatment stimulated a stronger induction of CD4+ T and CD8+ CTL cells compared to S2 alone. Collectively, these data provided a preliminary and theoretical basis for developing a novel vaccine against brucellosis.

## 2. Materials and Methods

### 2.1. Preparation of Ag85a-S2, Ag85a, and S2

The S2 vaccine was purchased from Qilu Animal Health Co., Ltd., with approval number 2015-150257011. For preparation of the Ag85a-S2, a previously reported broad-host-range plasmid pBEV was synthesized [31] by Tsingke Biotech (Beijing, China) and used as the backbone to express the Ag85a antigen in *B. suis* strain S2. The coding sequence of Ag85a was amplified from our previous plasmid pET28a-Ag85a [20], subcloned into pBEV, and verified by DNA sequencing. This plasmid was named pBEV-Ag85a. A total of 1 µL of 0.5 µg/µL pBEV-Ag85a was added into the 100 µL of the homemade B. suis S2 competent cells. The cell–DNA mixture was added between electrodes separated by 0.5 cm. The electroporation settings were: 650 V for 10 ms, 0.4 µF. The electroporated cells were transferred to a 15 mL tube containing 1 mL SOC-B media. The cells were incubated for 24 h at 37 °C, and 250 µL aliquots of cell culture was transferred onto trypticase soy broth (TSB) agar plates containing carbenicillin (50 µg/mL). The clones were sent for Sanger DNA sequencing. The *Brucella* suis S2 bacteria clone containing pBEV-Ag85a was named Ag85a-S2. The colony-forming units (CFU) of Ag85a-S2 and S2 vaccine were calculated by dilution of the sample and plating of several dilutions on TSB agar plates. The Ag85a used in this study was the purified endotoxin-free plasmid DNA pBEV-Ag85a.

### 2.2. Cell Culture

U937 (Cat. No.: CRL-1593.2) and Caco-2 (Cat. No.: HTB-37) cells were purchased from ATCC. U937 cells were cultured in RPMI-1640 medium supplemented with 10% fetal bovine serum. Caco-2 cells were cultured in Eagle’s minimum essential medium supplemented with 20% fetal bovine serum. All cells were in the 37°C humid CO_2_ incubators supplemented with 5% CO_2_.

As described previously, IEC (intestinal epithelial cells) and IEL (intraepithelial lymphocytes) were isolated [32]. For bone marrow-derived macrophage (BMDM) isolation, the protocol was from a recent report [33]. Briefly, for isolation of IECs, intestines were recovered and placed in ice-cold Hank’s balanced salt solution (HBSS). Mesenteric fat and external mucus were removed from intestines. The duodenal tract was harvested, opened longitudinally, cut into 1–2 mm pieces, and washed in ice-cold HBSS. Tissue pieces were digested for 30 min at 37 °C with Dispase (Sigma, St. Louis, MI, USA, Catalog #4818) and then centrifuged at 100× *g* for 3 min and resuspended in 37 °C DMEM medium. The pellet was resuspended and filtered through a 200 µm cell strainer and then reversely filtered using a 40 µm cell strainer to recover the >40 µm fraction. The recovered aggregates were then resuspended in DMEM and plated in 24 wells coated with 0.1 mg/mL Matrigel^®^ matrix. Cells were grown at 37 °C in a humidified atmosphere containing 5% CO_2_ and 95% air. After 24 h, the culture was washed with HBSS to remove unattached and dead cells, and the foci of proliferating enterocytes were replenished with DMEM supplemented with CHIR99021 (GSK3 inhibitor) and Epithelial Cell Growth Supplement (ScienCell, Carlsbad, CA, USA, Catalog #4152). For isolation of IELs, the small intestine was dissected and washed three times with ice-cold HBSS by inverting the tube 10 times. The supernatant was discarded. Intestine pieces were incubated with 20 mL of prewarmed dithioerythritol solution (HBSS supplemented with 10% FBS and 1 mM dithioerythritol) in a 50-mL siliconized Erlenmeyer flask containing a stir bar. The samples were stirred at 37 °C and 220 rpm on a magnetic stirrer for 20 min. The tissue and solution were transferred back to a 50 mL conical tube and vortexed for 10 s. The supernatant was collected into a new 50 mL conical tube through a 70 µm cell strainer. The cells in the supernatant were further pelleted by centrifuging at 400× *g* for 5 min at 4 °C and then resuspended in 8 mL Lymphoprep™ (STEMCELL, Vancouver, BC, Canada, Cat. No.: 07801). The 8 mL Lymphoprep™/cell suspension was transferred into a 15 mL polypropylene round-bottom tube. The tubes were centrifuged at 1600× *g* for 20 min. The top layer of the gradient was removed. The IELs from buffy coat at the interface were harvested to a 50 mL conical tube containing 40 mL cold DMEM media.

### 2.3. cGAS Knockout Cell Line Construction

Three gRNAs targeting the genomic region near the cGAS translation start site were designed by the online website (https://portals.broadinstitute.org/gppx/crispick/public, accessed on 11 May 2020). Three complementary gRNA pairs were annealed and ligated into pX330A-1×2 (Addgene, 58766) by a quick ligation ligase (NEB, M2200). We named these plasmids pX330A-cGAS_1/2/3. T7 endonuclease I assay was used to test the genomic editing efficacy of the 3 gRNAs. pX330A-cGAS_3 exhibited the highest editing efficacy and was used for further construction of cGAS KO cell line. To generate a cGAS knockout cell line, 1 µg of the pX330A-cGAS_3 plasmid was cotransfected into Caco-2 cells, and 200 µL of cells (5 cells/mL) were seeded onto 96-well plates 72 h post-transfection. The genomic DNA samples of single-cell colonies from 96-well plates were subsequently extracted using QuickExtract™ DNA Extraction Solution (Epicenter, QE09050), amplified by EasyTaq^®^ 2× Super Mix (Transgene, Beijing, China, AS111), and resolved on 1.5% agarose. The mutation of the cGAS locus was further validated by DNA sequencing. The successful knockout of cGAS was validated by Western blotting.

### 2.4. In Vitro Stimulation

Next, 2 × 10^5^ cells were plated in the 6-well plate in 2 mL culture medium without antibiotics. Cells were stimulated with 200 µL of PBS (control), 200 µL of 5 × 10^4^ CFU/mL S2 or Ag85a-S2, or 200 µL of 10 ng/µL Ag85a protein for 24 h. The supernatant or cell lysates were then collected for ELISA or qPCR analysis. The multiplicity of infection (MOI) is ~0.05 for S2 and Ag85a-S2.

### 2.5. Animal Immunization

The 5-week-old male C57BL/6 mice were randomly divided into 4 groups: the Ag85a-S2 group, the S2 group, the Ag85a group, and the control (PBS saline) group. Each group contained 8 mice. The mice received intragastric administration 3 times with 0.2 mL of 3 × 10^5^ CFU/mL Ag85a-S2 and S2, 0.2 mL of 0.9% NaCl, or 0.2 mL of 0.1 µg/µL Ag85a protein. The interval between the first 2 immunizations was 10 days, and the interval between the third immunization and the second immunization was 14 days. The mice were terminated two months after the last vaccination. IECs, IELs, serum, and whole blood were collected for ELISA or qPCR experiments. The experiment was repeated independently three times.

### 2.6. siRNA Transfection

Following this, 2 × 10^5^ freshly made IECs were plated in a 6-well plate 4 h before transfection, and 6 pmol RNAi duplexes against STING or cGAS were diluted in 50 µL Opti-MEM ^®^ I Reduced Serum Medium. A total of 1 µL of Lipofectamine ™ RNAiMAX was gently diluted in 50 µL of Opti-MEM ^®^ I Reduced Serum Medium. The diluted RNAi duplexes and Lipofectamine ™ RNAiMAX were incubated together for 10 min and then added to each well-containing cell. The siRNA duplexes against cGAS or STING were synthesized by GenePharma Inc., Suzhou, China. The siRNAs with 3′ dTdT modification targeted the following sequences: cGAS 5′-ccagcagcaggccauccug-3′; STING 5′-ggccagccugaugauccuu-3′.

### 2.7. qPCR Assay

Total RNA was extracted using TRIzol reagent (Thermo, Waltham, MA, USA, 15596026), according to the manufacturer’s instruction. The cDNA synthesis was performed by Revert Aid first strand cDNA synthesis kit (Thermo, K1622) using random primers. The cDNA was diluted 5-fold in DNase-free water and further used for qPCR analysis. Altogether, 1 µL of cDNA, 2.5 µL of forward primer, 2.5 µL of reverse primer, 19 µL of water, and 25 µL of qPCR 2× supermix (Transgen, Beijing, China, AQ131-01) were mixed together. PCR reaction was performed on a Biorad CFX96 real-time PCR detection system, and 2^−ΔΔCT^ method was used for quantification.

### 2.8. Western Blotting Assay

Next, 1 × 10^6^ cells were lysed in 200 µL of RIPA lysis buffer (Thermo Fisher, Cat. No.: 89900), supplemented with 2 µL of 100× protease inhibitor cocktail (Abcam, Cat. No.: ab271306). Then, 10 µg of protein from cell lysates of wild-type or cGAS^−/−^ Caco-2 cells were loaded to the precast NuPAGETM gels (Thermo, NP0321BOX) and separated by electrophoresis. The proteins were further transferred to 0.45 µm PVDF membrane. The transfer buffer (25 mM Tris-HCl pH 8.0, 120 mM glycine supplemented with 20% (*v*/*v*) methanol) was cooled down to 4 °C in the refrigerator. The transfer conditions were: current 200 mA and time 90 min; transfer cassette was put on ice. The membrane was blocked by 5% non-fat milk in PBS for 2 h at 25 °C. The membrane was further incubated with rabbit polyclonal cGAS primary antibody (Abcam, Cambridge, UK, ab224144) at a dilution of 1:1000 overnight at 4 °C. The membrane was further washed three times with PBS and incubated with HRP-conjugated goat anti-rabbit secondary antibody (Abcam, ab205718) at a dilution of 1:5000 and incubated for 1 h at 25 °C. The PVDF membrane was washed three times with PBS and developed by Pierce™ ECL Western Blotting Substrate (Thermo, A38554) and captured by Tanon chemidoc system.

### 2.9. ELISA Assay

The cGAMP (Cat. No.: EIAGAMP) and IFNα (Cat. No.: BMS6027) ELISA kits were purchased from Invitrogen. The IFIT1 ELISA kit (Cat. No.: OKEH06734) was purchased from Aviva Systems Biology. The *Brucella* IgG ELISA was from IBL (Cat. No.: RE56841). All the assays were performed according to the manufacturers’ instructions. In brief, 2 × 10^5^ cells were collected and lysed by ultrasonic homogenizers with microtips in 1 mL PBS freshly supplemented with 1×protease cocktail. Some 50 µL of cell lysates aliquots were used for downstream ELISA assay for IFNα, cGAMP, and IFIT1. For *Brucella* IgG ELISA, 50 µL of serum samples were collected from mice two months after last vaccination, and 2 × 10^4^ cells were seeded into the 96-well plates 12 h prior to stimulation. The cells were stimulated as described previously, and the supernatants were collected for IFNα ELISA. A total of 50 µL of the serum from vaccinated mice were collected for *Brucella* IgG ELISA assay.

### 2.10. RNA Sequencing and Bioinformatics Analysis

Total RNA was extracted from wild-type and cGAS-KO Caco-2 cells. The cDNA libraries were constructed for each pooled RNA sample using the VAHTS^TM^ Total RNA-seq according to the manufacturer’s instructions. In brief, mRNA was purified from 1 µg of total RNA by mRNA capture beads and fragmented by heating 5 min at 94 °C in the presence of Mg^2+^. The first and second strand of cDNA was synthesized using the fragmented mRNA as template. The cDNA was further processed with end repair, addition of dA-tails, and ligation of adapter. The cDNA was amplified to construct the library. The raw sequencing data were also evaluated by FAST-QC, including quality distribution of nucleotides, position-specific sequencing quality, GC content, the proportion of PCR duplication, and k-mer frequency. We used DESeq2.0 to analyze the differential genes between cGAS-KO and wild-type group. The parameters were: |log2FC| > 1 and *p* value < 0.05. Pathway analysis was used to determine the differential genes’ significant pathways. Pathway annotations of microarray genes were downloaded from KEGG (http://www.genome.jp/Kegg/, accessed on 10 July 2021). A Fisher exact test was used to find the significant enrichment pathway. The resulting *p* values were adjusted using the BH FDR algorithm. Pathway categories with an FDR < 0.05 were reported.

### 2.11. Statistical Analysis

Statistical analysis was carried out as previously described [34]. In brief, one-way ANOVA Dunnett’s multiple comparison test was used for comparing the multiple sets of data by using Prism 8 (GraphPad Software Inc., San Diego, CA, USA). A *p* value of less than 0.05 was significant. Statistical significance levels are denoted as follows: * *p* < 0.05; ** *p* < 0.01.

## 3. Results

### 3.1. Ag85a-S2 Upregulates the Key Components in the cGAS-STING Cascade

**Figure 1:**
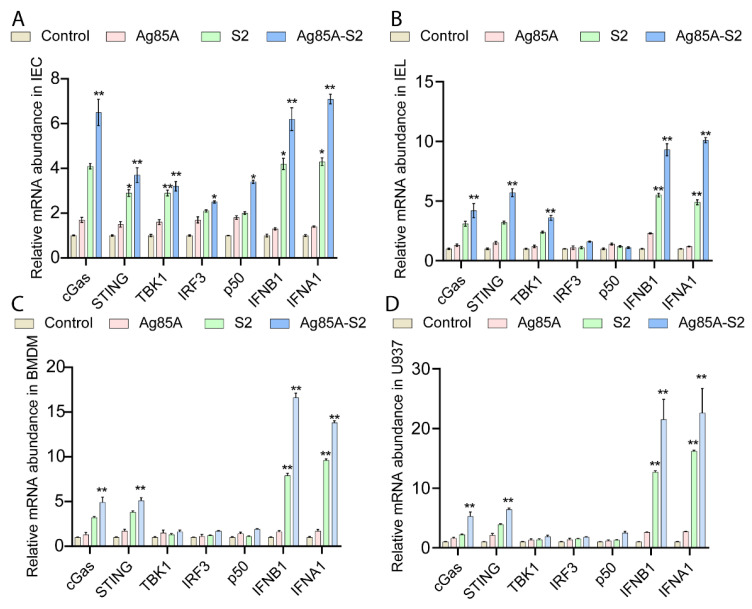
Ag85a-S2 upregulates the mRNA abundance of key components in the cGAS-STING signaling pathway both in vivo and in vitro. (**A**,**B**) A total of 20 mice in each group were orally administered with PBS (control), S2, Ag85a, or Ag85a-S2. IECs and IELs were isolated after last immunization. mRNA expression levels of cGAS key components were detected in IEC (**A**) and IEL (**B**) by RT-qPCR. IECs and IELs were obtained two months post-oral administration. (**C**,**D**) BMDM (**C**) or U937 (D) cells were stimulated with PBS (control), S2, Ag85a, or Ag85a-S2 for 4 h, and transcripts levels were measured by RT-qPCR. mRNA levels were normalized to the control group. GAPDH was used as the reference gene for normalization. Data are presented as mean ± S.E.M. of three independent experiments. Post hoc test in ANOVA was used to calculate the significance between control, S2, Ag85a, and Ag85a-S2 group. *, *p* < 0.05; **, *p* < 0.01.

To test whether Ag85a-S2 boosted immune response via the cGAS-STING pathway, we tested multiple key molecules in the cGAS signaling pathway in intestinal epithelial cells (IEC) or intraepithelial lymphocytes (IEL) after oral administration of normal saline, S2 vaccine, Ag85a, and the Ag85a-S2 developed in our laboratory. Here, we showed that the transcript levels of cGAS, STING, TBK1, IFNB1, and IFNA1 were significantly upregulated in IEC (Figure 1A) and IEL (Figure 1B) after stimulation with Ag85a-S2 and S2. In contrast, the above transcripts only increased marginally or remained unchanged in the control (PBS saline treatment) and Ag85a groups. Moreover, the cGAS-STING activation was stronger in Ag85a-S2 group than that in the S2 alone group. Similar results were recapitulated in vitro in bone marrow-derived macrophage (BMDM) (Figure 1C) and U937 cell line (Figure 1D).

**Figure 2:**
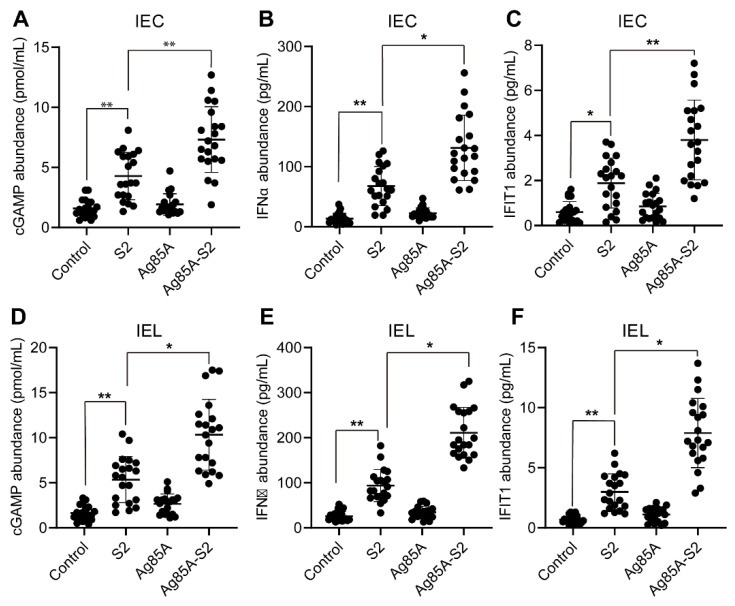
cGAS-STING downstream targets are significantly upregulated upon Ag85a-S2 treatment. (**A**–**F**) The downstream targets regulated by cGAS-STING were measured by ELISA. Twenty mice in each group were orally administered with PBS (control), S2, Ag85a, or Ag85a-S2. IEC (**A**–**C**) and IEL (**D**–**F**) cells were collected and subjected to ELISA. The cGAMP (**A**,**D**), IFNα (**B**,**E**), and IFIT1 (**C**,**F**) in the cell lysates from IEC (**A**–**C**) and IEL (**D**–**F**) were measured. *, *p* < 0.05; **, *p* < 0.01, one-way ANOVA Friedman test with Dunn’s multiple comparisons post-test.

To further determine the upregulation of key gene transcripts in the cGAS signaling pathway, we used ELISA to measure three downstream effector cytokines of the cGAS-STING signaling pathway in IEC and IEL cells after oral administration, including cGAMP, IFNα, and IFIT1. We showed that the protein abundance of cGAMP, IFNα, and IFIT1 were significantly increased in IEC (Figure 2A–C) and IEL (Figure 2D–F) after oral administration of the Ag85a-S2, whereas normal saline and Ag85a alone failed to alter the abundance of the cytokines. Although S2 can activate the expression of the above three cytokines, its activation degree is milder than that of Ag85a-S2. Collectively, these data suggested that Ag85a-S2 could be more potent for the immune response, at least partially through the cGAS-STING pathway, compared with S2 alone.

### 3.2. Generation of cGAS Knockout Cell Line

**Figure 3:**
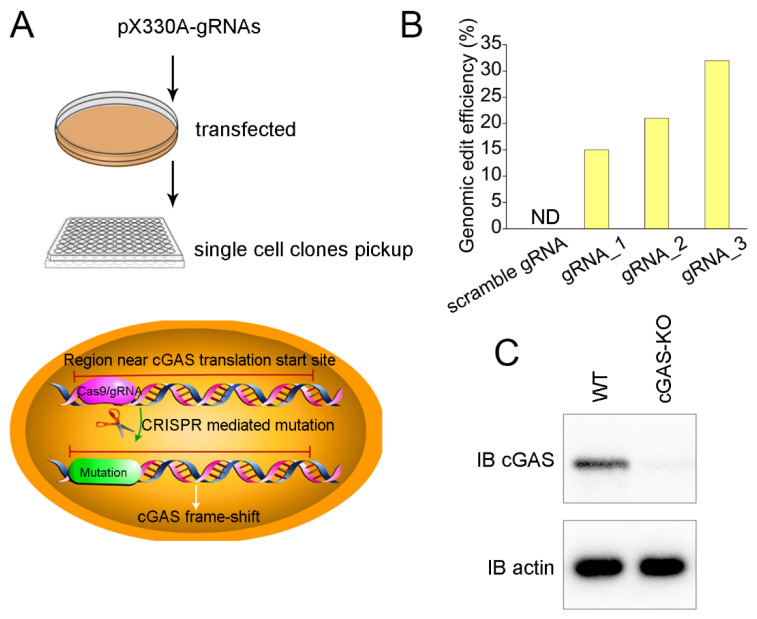
Construction of cGAS knockout Caco-2 cells by CRISPR. (**A**) Schematic presentation of CRISPR-mediated point mutation. Cells were transfected with a pX330 plasmid encoding gRNAs targeting the region near cGAS translation and spCas9. Single-cell colonies were picked up for Sanger sequencing validation. (**B**) Detection of cutting efficiency of different gRNA. Caco-2 cells were transfected with pX330A plasmids encoding different gRNAs and spCas9. Genomic DNA was extracted, and the genomic regions containing the cutting sites were PCR amplified and subjected to T7 endonuclease I assay. (**C**) The single-cell colony carrying homozygous deletion of cGAS (cGAS-KO) was verified by Western blotting. N.D.—not detected.

To further confirm the results obtained in the in vivo mice model, we used CRISPR technology to introduce point mutations at the translation start site of cGAS and screened out the homozygotes that lost the expression of cGAS by picking up single-cell clones (Figure 3A). We chose Caco-2 for this experiment. Caco-2 is an immortalized cell line of human colorectal adenocarcinoma cells. It is primarily used as a model of the intestinal epithelial barrier. Thus, we designed three gRNAs targeting the translation start site of cGAS and estimated the cleavage efficiency of different gRNAs by Sanger sequencing. The results showed that the efficiency of gRNA3 was better than that of gRNA1 and gRNA2 (Figure 3B). Caco-2 cells were transfected with Cas9/gRNA3. After screening 158 single-cell clones, we successfully obtained a cell line with homozygous deletion of cGAS (cGAS-KO hereafter), which was validated by Western blotting (Figure 3C). 

We then performed next-generation sequencing on the cGAS-KO and wild-type Caco-2 cells. RNA-seq results showed that there was a total of 532 differentially expressed genes in the cGAS-KO cells compared to those in the wild-type Caco2 cells. A total of 274 and 258 out of the 532 genes were upregulated or downregulated, respectively (Supplementary Figure S1A). We analyzed the distribution of overall reads and found that cGAS-KO did not alter the distribution of reads (Supplementary Figure S1B). We performed a signaling pathway enrichment analysis on the 532 differential genes. The results showed that differentially expressed genes in cGAS-KO cells were mainly enriched in the following pathways: the interferon I signaling pathway, defense response to the virus, and other immune responses involving cytokines (Supplementary Figure S1C). Several representative genes that were related to the interferon I signaling pathway were shown (Supplementary Figure S1D). The above results suggest that the knockout of cGAS could trigger changes in multiple immune-related signaling pathways, which probably compensated for the lack of cGAS. In addition, these results also suggested that the cGAS-KO cell line we constructed met its expected biological functions.

### 3.3. Depletion of cGAS-STING Signaling Impairs Immune Cytokines Secretion

**Figure 4:**
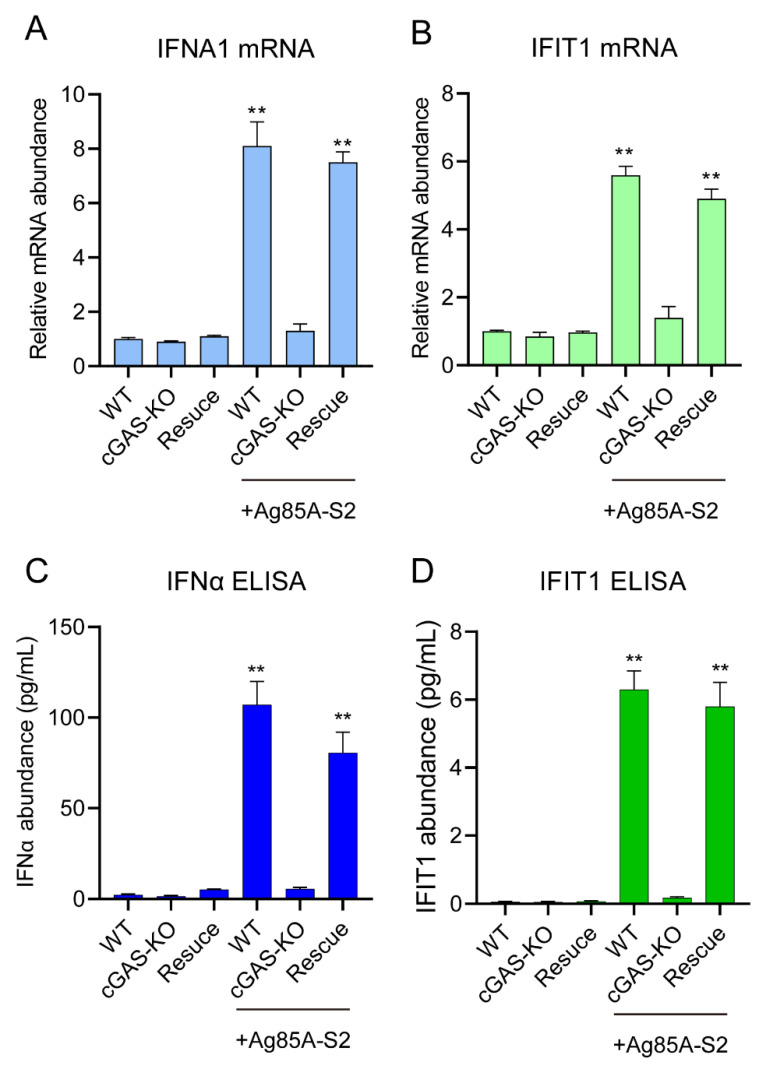
cGAS-STING signaling axis senses the Ag85a-S2 stimulation. (**A**–**D**) Quantification of mRNA levels (**A**,**B**) and secreted protein abundance (**C**,**D**) of IFNA1 and IFIT1 upon Ag85a-S2 stimulation in wild-type (WT), cGAS knockout (cGAS-KO), or cGAS knockout rescued with HA-cGAS (rescue) Caco-2 cells. GAPDH was used as the reference gene for normalization. For rescue experiment, cGAS knockout cells were stably transduced with full-length HA-cGAS. **, *p* < 0.01, one-way ANOVA Friedman test with Dunn’s multiple comparisons post-test.

Furthermore, we stimulated wild-type, cGAS-KO, and rescued cell lines (i.e., cGAS-KO cell lines transduced by full-length cGAS) with Ag85a-S2 and simultaneously analyzed the protein and transcript levels of IFNα and IFIT1. We showed that Ag85a-S2 stimulation significantly increased the protein and transcript levels of IFNα and IFIT1 in the wild-type cells (Figure 4A–D) but failed to increase the protein and transcript levels of IFNα and IFIT1 in the cGAS-KO cells (Figure 4A–D). In addition, we rescued the cGAS-KO cell with full-length cGAS. The rescue procedures did not alter the protein and transcript levels of IFNα and IFIT under the resting state but significantly upregulated the expression of IFNα and IFIT1 upon Ag85a-S2 stimulation (Figure 4A–D).

**Figure 5:**
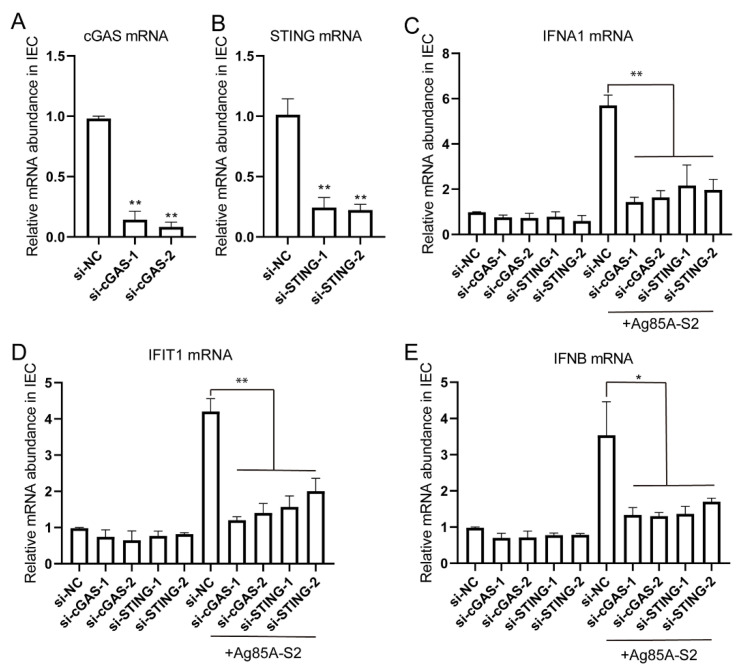
Silencing of cGAS or STING inhibited the immune response triggered by Ag85a-S2. (**A**–**D**) Naïve IECs were transfected with 10 nM siRNA duplexes against cGAS or STING; 72 h post-transfection, naïve IECs were stimulated with Ag85a-S2 for 24 h. The relative mRNA level of cGAS (**A**), STING (**B**), IFNA1 (**C**), IFIT1 (**D**), and IFNB (**E**) was quantified. Gapdh was used as the housekeeping gene. Data are presented as mean ± S.E.M. of three independent experiments. ** *p* < 0.01; *, *p* < 0.01, one-way ANOVA Friedman test with Dunn’s multiple comparisons post-test.

To further test whether this phenotype could be recapitulated, we isolated IEC with a purity of around 95% and silenced cGAS and STING in IEC. The knockdown efficiencies of cGAS and STING were verified by RT-qPCR (Figure 5A,B). As expected, the depletion of either cGAS or STING attenuated the IFNα, IFIT1, and IFNB expression levels after Ag85a-S2 stimulation (Figure 5C–E). Altogether, our in vivo results confirmed that the intestinal cells responded to Ag85a-S2 through the cGAS signaling pathway.

### 3.4. Ag85a-S2 Triggers Cellular Immune Response

**Figure 6:**
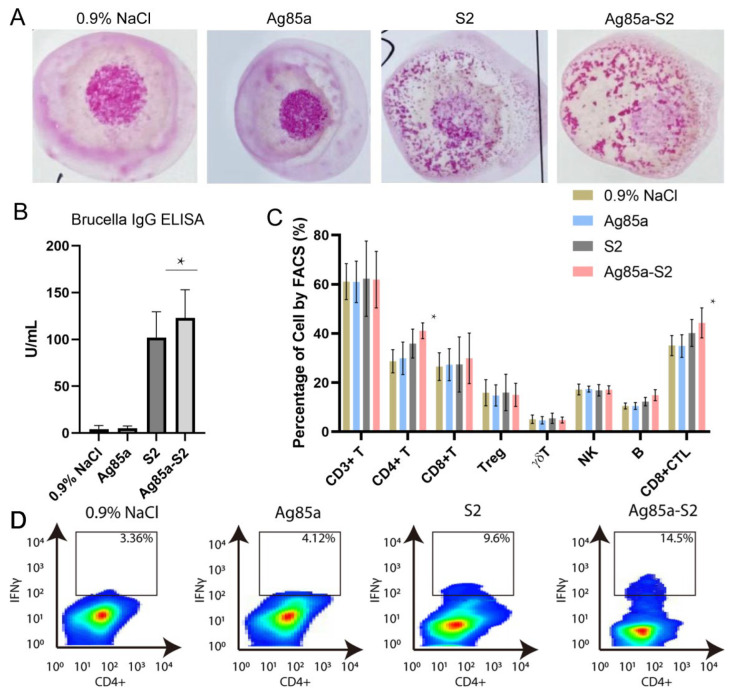
Ag85a-S2 promotes Brucella-specific IgG level and cellular immunity. (**A**) Representative pictures illustrating the RBT assays of serum from 0.9% NaCl-, Ag85a-, S2-, or Ag85a-S2-treated mice. (**B**) Quantification of serum Brucella IgG level in the serum as indicated. (**C**) Detection of lymphocyte subpopulations in the blood from 0.9% NaCl-, Ag85a-, S2-, or Ag85a-S2-treated mice. Blood and serum were sampled two months after the lase immunization. (**D**) FACS analysis of IFNγ-expressing IELs in the intestinal mucosa derived from mice vaccinated with 0.9% NaCl, Ag85a, S2, or Ag85a-S2. * *p* < 0.05. one-way ANOVA Friedman test with Dunn’s multiple comparisons post-test.

*Brucella* are generally regarded as intracellular pathogens. Protection against intracellular bacteria requires long-lasting immune responses and adaptive cellular immunity. The Rose Bengal test is a rapid agglutination reaction using *Brucella* as a bacterial suspension, stained with Rose Bengal in a buffered acid medium. After mixing equal parts of Rose Bengal antigen and serum, the appearance of colored agglutinations is observed in the case of brucellosis. The sensitivity limit is 25 IU/mL. The test highlights IgG and is positive later (2 to 3 weeks), and the sensitivity of the technique is at least 95%. Thus, we used the Rose Bengal test (RBT) to detect the anti-*Brucella* antibody in the mice serums stimulated with Ag85a, Ag85a-S2, S2, and 0.9% NaCl. As shown in Figure 6A, both S2 and Ag85a-S2 showed positive results. We further applied ELISA to quantify the *Brucella*-specific IgG level. As shown in Figure 6B, the IgG level in the Ag85a-S2 and S2 groups were significantly higher than the Ag85a and 0.9% NaCl group. Although Brucella-specific antibodies have been shown to have a limited role during *Brucella* infection, we indeed observed a mild (less than 10%) upregulation of *Brucella*-specific IgG level in Ag85a-S2 compared to that in S2 (Figure 6B).

Cell-mediated immunity, which entails the activation of the bactericidal mechanisms of antigen-presenting cells (macrophages and dendritic cells) and the subsequent development of antigen-specific CD4+ T and CD8+ cytotoxic T lymphocyte (CTL) cell clones, is necessary for resistance against intracellular bacterial infections. Thus, we tested the lymphocyte subpopulations in the blood of mice stimulated with Ag85a, Ag85a-S2, S2, and 0.9% NaCl. Although both Ag85a-S2 and S2 could increase the abundance of CD4+ T and CD8+ CTL cells, Ag85a-S2 triggered a stronger upregulation of the above two types of cells compared with S2 alone, whereas Ag85a and 0.9% NaCl failed to do so (Figure 6C). In addition, the host immune reaction is typically represented by immune cell infiltration. Thus, we tested the TH1 cell population of IELs after challenging with Ag85a-S2. We found that the percentage of IFNγ-expressing TH1 population in the Ag85a-S2 group was higher than that in the S2 alone group. This piece of data suggested that a stronger TH1 immune cell infiltration caused by Ag85a-S2 might contribute to better protection against *Brucella* (Figure 6D). Collectively, these data suggested that the stimulation with Ag85a-S2 led to a more potent cellular immune response and acquired immunity compared to S2 alone.

## 4. Discussion

*Brucella* can escape the immune monitoring in the following ways: First, the *Brucella* surface lacks conserved PAMPs, such as capsule, fimbriae, and cilia, that can effectively activate the innate immune response. Second, a distinctive feature of *Brucella* is that it contains atypical lipopolysaccharide (LPS), which is a weak TLR4 agonist and reduces its recognition by TLRs. Third, by reducing, modifying, or hiding PAMPs [35], *Brucella* enters the macrophage phagosome through lipid rafts and then fuses with the endoplasmic reticulum to form *Brucella*-containing vacuoles (BCVs) [36]. BCVs do not fuse with lysosomes and escape bacterial degradation by lysosomal enzymes, thereby achieving intracellular survival and replication. Finally, Btp1/TcpB protein from *Brucella* can activate the signaling pathway by inhibiting the immune response mediated by TLRs, resulting in the persistent infection of *Brucella* in vivo [37]. Therefore, the naturally live-attenuated *Brucella* could not trigger an effective immune response.

The rational development of new and improved vaccines requires a comprehensive understanding of innate immune activation. A previous report suggested that the vaccine adjuvant chitosan activated the cGAS-STING pathway, mediating the selective production of type I IFN and interferon-stimulated genes. These cytokines mediated the activation and maturation of DCs and Th1 cell response [38]. A recent review highlighted that vaccines adjuvant with cGAS-STING agonists were found to mediate a robust immune defense against infections and cancer [39]. The cGAS-STING signaling pathway was also involved in maintaining gut homeostasis and played a protective effect in controlling gut inflammation [40,41,42]. Thus, the activation of the cGAS-STING pathway can elicit beneficial vaccine outcomes. We and others have demonstrated the potential use of Ag85a in the activation and modulation of immune responses [18,19,20,43]. Thus, Ag85a is being utilized as a tool in the construction of new vaccines, such as recombinant attenuated vaccines, DNA vaccines, and subunit vaccines. Here, we found that Ag85a-S2 rather than Ag85a alone triggered an efficient cGAS-STING signaling pathway. *Brucella*, including live-attenuated S2 and its derivative Ag85a-S2 developed here, is a facultative intracellular parasitic bacterium. During intracellular bacterial infections, Brucella DNA is probably sensed via the cGAS-STING signaling pathway as a major bacterial component. This might provide a sound explanation for the activation of the cGAS-STING signaling pathway. In addition, the shelf life of the current commercial S2 vaccine is approximately 12 months at 4 °C. The activation of the immune response at the cellular level is comparable between the freshly prepared Ag85a-S2 and the old ones, which is kept after 12 months of storage at 4 °C. Therefore, the shelf life of this Ag85a-S2 was at least 1 year at 4 °C. Collectively, these results suggest that the Ag85a-S2 may provide more effective protection against *Brucella*, thereby reducing the dosage compared to the traditional S2 vaccine. The reduction of the S2 dosage will help decrease the risk of vaccine toxicity.

When *Brucella* invades the host, it first reaches the lymph nodes with the lymphatic fluid and colonizes the cells for amplification, forming local pathological foci. When the number of Brucella reaches a certain level, the cells are destroyed, and Brucella distributes to multiple organs throughout the body with the blood flow to colonize and form multiple lesions. Therefore, brucellosis is a multiorgan systematic disease, which can impair multiple systems, such as the motor system, nervous system, digestive system, cardiovascular system, genitourinary system, endocrine system, and respiratory system, etc., and exhibit different symptoms [44]. Currently, in order to study the effectiveness of the vaccine candidate against Brucella, researchers typically infect vaccinated animals with wild-type Brucella strains and dissect spleen and lung tissues after a certain period of infection to determine the colony formation of Brucella. Although the colony formation assays partially reflect the protection efficacy against Brucella after vaccination, this method cannot be applied to natural live-attenuated vaccines because naturally live-attenuated vaccines are in fact a type of Brucella and can cause systemic infections. The widely used colony formation assays cannot distinguish between naturally attenuated and wild-type Brucella strains. In conclusion, there are no protocols available to accurately and comprehensively detect changes in the number of bacteria infected with wild-type Brucella after natural live-attenuated vaccination. Currently, we have not tested the bacterial reduction in live animals after vaccination.

## Data Availability

All the RNA-seq raw data and processed files for this paper have been deposited to Gene Expression Omnibus under the following accession: GSE205786.

## Supplementary Materials

The following supporting information can be downloaded at: https://www.mdpi.com/article/10.3390/vaccines10122170/s1, Figure S1: cGAS regulates key genes in IFN and cytokines pathways.
