# Supplementary material for: Ag85a-S2 Activates cGAS-STING Signaling Pathway in Intestinal Mucosal Cells
Source: Vaccines (Basel). 2022 Dec 16;10(12):2170. doi: 10.3390/vaccines10122170 (PMC9785823; doi:10.3390/vaccines10122170)
Supplement: Supplementary file 1 [file vaccines-10-02170-s001.zip › vaccines-2066318-supplementary.pdf]

vaccines

MDPI

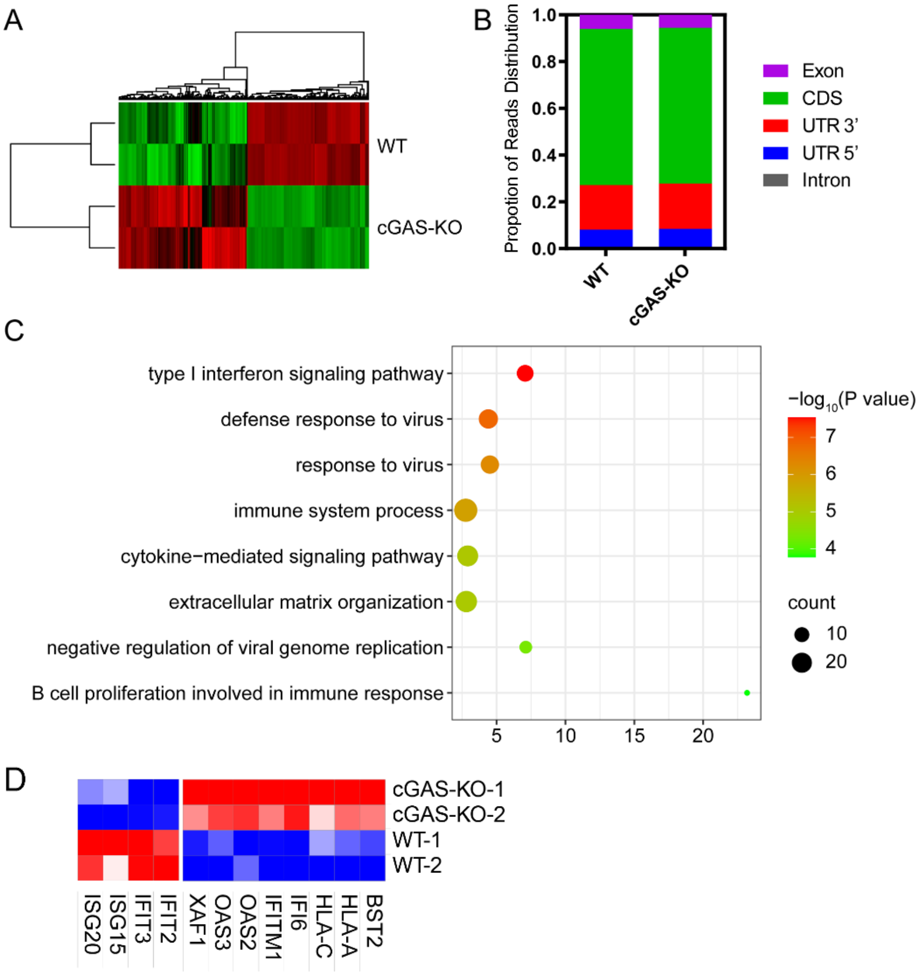

Figure S1. cGAS regulates key genes in IFN and cytokines pathways. (A) Differentially expressed genes in wild-type (WT) and cGAS knockout Caco-2 cells (cGAS-KO). (B) The distribution of reads from RNA-seq across the gene structure. (C) Gene ontology analysis of differentially expressed genes in (A). (D) Sub-heatmap showing the representative differently expressed genes.
